# Multispecialty surgical management of large clavicular tumor: A case report

**DOI:** 10.1016/j.ijscr.2022.107375

**Published:** 2022-07-06

**Authors:** Mohammad Aladaileh, Tom Moran, Austin Duffy, Karen Redmond, David O'Briain

**Affiliations:** a Thoracic Surgery Department, The Mater Misericordiae University Hospital, University College of Dublin, Dublin, Ireland; b Paediatric and Adult ENT, The Mater Private Hospital, Dublin, Ireland; c Department of Oncology, The Mater Misericordiae University Hospital, Dublin, Ireland; d Department of Orthopaedic Surgery, The Mater Misericordiae University Hospital, Dublin, Ireland

**Keywords:** Clavicular mass, Carcinoma, Partial claviculectomy, Multispecialty approach

## Abstract

**Introduction:**

Primary and metastatic malignancies of the clavicle are extremely uncommon and difficult to treat. It carries a high morbidity rate. Total or partial claviculectomy is the recommended treatment option.

**Presentation of case:**

A 59-year-old male was admitted with a large left clavicular huge mass; biopsy showed poorly differentiated adenocarcinoma. Treatment started with chemoradiotherapy followed by complete excision of the tumor surgically.

**Discussion:**

For treating this rare, difficult case, partial claviculectomy performed by a multispecialty team integrating three surgical skill sets: thoracic, ENT, and orthopedic surgeons, yields the best results. Clavicular carcinoma of known primary (CUP) is rare, and chemoradiotherapy showed preferable results in management.

**Conclusion:**

MDT reviews with surgeons, radiologists, pathologists, oncologists, and radiation oncologists are required in rare and complex cases of clavicular malignancy. Multispecialty surgical involvement is required for the safe excision of clavicular tumors with appropriate oncologic margins and fewer complications.

## Highlights

• A huge Clavicular tumor is challenging and rare.
• Multiple specialty surgeons and physicians are needed for resection and treatment.
• Primary malignancy is rare, and Unknown primary clavicular tumor is extremely rare.

## 1 Introduction

The clavicle's primary and metastatic malignancies are very rare, with a reported incidence of 0.45 % to 1.01 % of all bone tumors [1]. A formal multidisciplinary team meeting is critical for case management. We present the case of a carcinoma of unknown primary (CUP) of the clavicle abutting the sternoclavicular joint requiring SC joint resection. Total or partial claviculectomy is the procedure of choice. A multispecialty team approach to surgery yields the best results for such cases.

Plasma cell tumors and osteochondromata are the most common types of clavicular malignant and benign tumors, respectively [2]. Clavicular carcinoma is a rare metastatic bone tumor. Metastatic tumors are frequently of insidious onset and typically affect males above 40-year-old. The main clinical features include pain and local mass effect. Partial or total resection of the clavicle is considered the optimal treatment option [3]. Clavicular carcinoma of unknown primary (CUP), as in our case, is extremely rare [4].

## 2 Case report

**Fig. 1:**
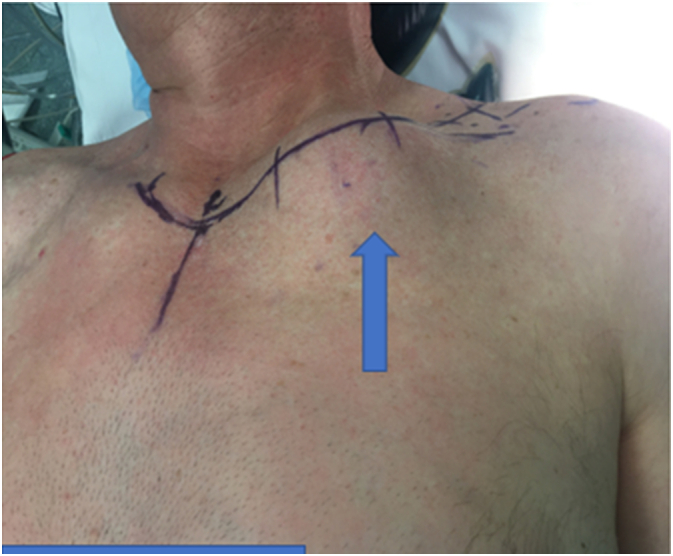
The curvilinear skin incision.

We present the case of a 59-year-old male ex-smoker with insidious onset of a left clavicular mass (6.5 × 5 cm) over six months (Fig. 1). He presented with left shoulder pain, he is a builder, and his BMI is 36 kg/m^2^. He had no neurological or vascular clinical signs or symptoms in the left upper limb. He has no family history of malignant diseases and is not on any chronic medications.

**Fig. 2:**
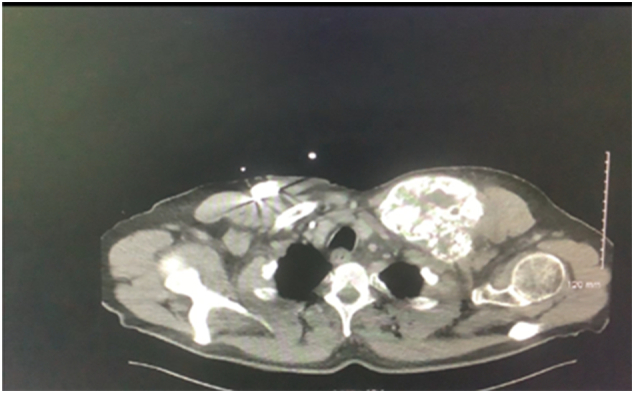
Contrast-enhanced CT-scan for left clavicular 10 × 8 cm.

**Fig. 3:**
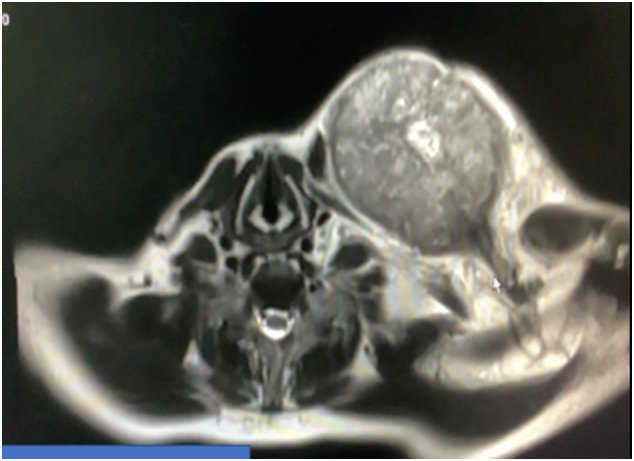
Contrast-enhanced MRI with huge clavicular mass.

Radiological investigations, including contrast-enhanced computed tomographic scan (CT-scan), magnetic resonance imaging (MRI), and a positron emission tomography CT scan (PET-Ct), revealed a solid mass extending from the middle third of the clavicle to the sternoclavicular joint (Fig. 2, Fig. 3). The external jugular vein was occluded on venous phase contrast scanning. A clear tissue plane appeared to be present between the lesion and all other neurovascular structures. The case was discussed at the multidisciplinary meeting (MDT), and a CT-guided biopsy was advised. The biopsy identified a poorly differentiated adenocarcinoma of unknown origin. The immuno-histochemical assessment reviewed by a musculoskeletal specialist pathologist revealed positive CEA and diffuse positivity for P16. The results were felt to favor a gastrointestinal primary. Upper and lower gastrointestinal endoscopy did not identify any suspicious lesions.

Given the negative investigations, the tumor mass was diagnosed as carcinoma of unknown primary (CUP) with local disease progression only with no regional or distant disease. The tumor was staged as a TxN0M1b oligometastasis.

This work has been reported in line with the SCARE 2020 criteria [6].

## 3 Management and follow-up

After a discussion with the patient, he agreed to go for chemoradiotherapy first, and the surgical option will be next. The patient received six cycles of FOLFOX chemotherapy and local radiotherapy, which was well tolerated. The mass had increased to 10 × 8 cm by the time of completion of neoadjuvant treatment.

A multispecialty approach involving thoracic, orthopedic, and ENT surgeons were employed for the partial claviculectomy.

**Fig. 4:**
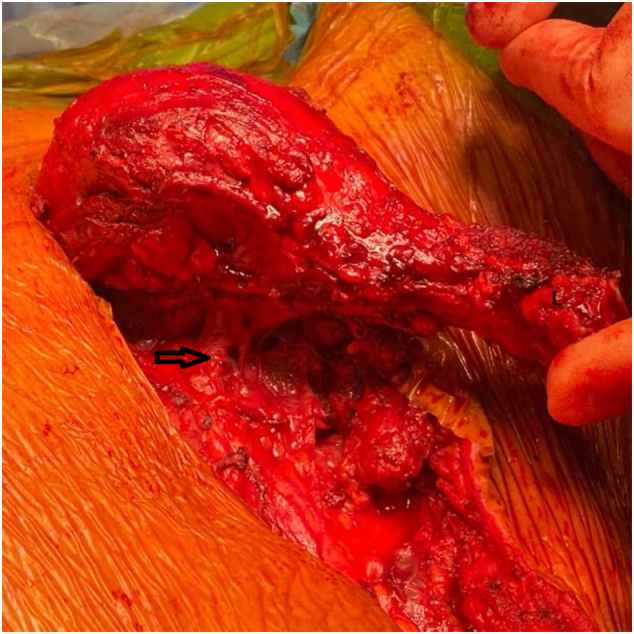
Intra-operative image for the tumor, the arrow indicates the inferior part of the external jugular vein.

**Fig. 5:**
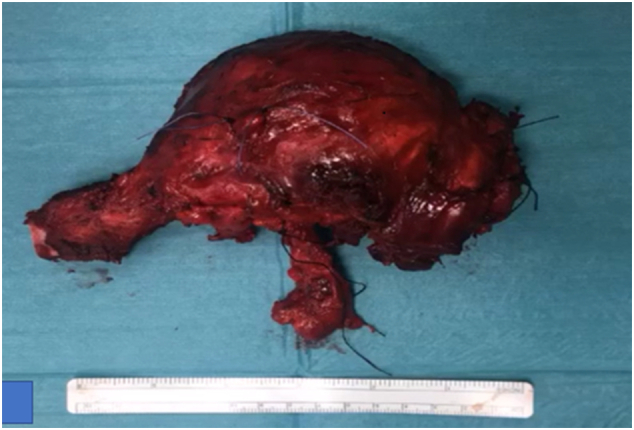
The specimen picture.

Under general anesthesia with IV antibiotic prophylaxis, the anatomic landmarks and incisions were planned and marked (Fig. 1). A curvilinear incision was described for the partial claviculectomy and extended proximally for unilateral sternoclavicular joint resection. Subplatysmal flaps were elevated. A safe oncologic margin was preserved superiorly, dividing the sternocleidomastoid muscle (SCM) and the superior part of the external jugular vein. The thoracic duct was noted to be adherent to the tumor mass and was ligated. Laterally the clavicle was exposed to the acromioclavicular joint. The distal clavicle was divided with an oscillating saw 5 cm from the acromioclavicular (AC) joint, preserving the AC joint, the coracoclavicular (CC) ligaments, the majority of the deltoid origin, and the clavicular insertion of the trapezius. Inferiorly the neurovascular structures were identified. The external jugular vein was noted to arise from the inferior part of the tumor (Fig. 4). It was divided using a vascular endo GIA stapler. The sternoclavicular joint was exposed and excised with a rim of manubrium using an oscillating saw. The subclavian vessels and the brachial plexus were identified and preserved, and the specimen was taken out (Fig. 5). Homeostasis was meticulously maintained. A Redivac drain was inserted. SCM sutured to the clavicular head of pectoralis major. The skin was not involved.

The patient had routine postoperative care, and his recovery was uneventful. He was discharged on postoperative day 4 with good pain control, a healthy wound, and no neurovascular deficit. Passive mobilization with progressive active-assisted rehabilitation was initiated two weeks after surgery.

The histopathology results showed Huvos grade 4 complete response to chemoradiotherapy, indicating the absence of active cancer cells in multiple high-powered fields.

A chest CT showed no evidence of tumor recurrence at one year of follow-up. Excellent active range of motion of the shoulder was noted with a Constant score of 98/100. There was no clinical evidence of recurrence nor any new clinical finding to suggest a potential primary source.

## 4 Discussion

Whether primary or metastatic, tumors involving the clavicle may require partial or total clavicular resection for local control or cure. The rarity of clavicular tumors, the pathological diagnosis of CUP, the size of the lesion, and the necessity for a multispecialty surgical approach highlight the importance of this case report. In addition, the tumor was invading the neck structures and encasing the external jugular vein, which makes our case unique because we describe the surgical approach for such challenging cases.

Clavicular reconstruction has been performed with allograft, autograft, and mega-prosthesis variations, none of which reliably led to clinical outcomes superior to isolated partial or total claviculectomy. Excision of the clavicle without reconstruction was associated with faster rehabilitation and a lower risk of further surgery [5].

Carcinoma of unknown primary (CUP) accounts for about 3–5 % of all malignancies. CUP is associated with increased morbidity and mortality as it is, by definition, metastatic at the time of diagnosis, with a median survival between 8 weeks and 24 months [4]. The majority of CUP cases have an infraclavicular tissue of origin, with a preference for a gastrointestinal tract source, as suggested by immunohistochemical assessment in the described case.

Surgical management in these cases requires a detailed surgical plan and a multidisciplinary team approach. The complex anatomy of the clavicle and its surrounding structures, such as the brachial plexus, thoracic duct, subclavian artery, subclavian vein, innominate vein, carotid artery pleura, and lung bridges the surgical comfort zones of cardiothoracic surgeons, ENT surgeons, and orthopedic surgeons.

## 5 Conclusion

For rare and complex cases of clavicular malignancy, MDT reviews with surgeons, radiologists, pathologists, oncologists, and radiation oncologists are necessary. Multispecialty surgical involvement is frequently required for the safe excision of clavicular tumors with appropriate oncologic margins.

## Sources of funding

No source of funding.

## Ethical approval

Not applicable.

## Consent

Written informed consent was obtained from the patient for publication of this case report and accompanying images. A copy of the written consent is available for review by the Editor-in-Chief of this journal on request.

## Research registration

Not applicable.

## Guarantor

Mohammad Aladaileh.

## Provenance and peer review

Not commissioned, externally peer-reviewed.

## CRediT authorship contribution statement

Mohammad Aladaileh: Analysis and interpretation of data, drafting the article, final approval of the version to be submitted.

Tom Moran: drafting the article, final approval of the version to be submitted.

Austin Duffy: diagnostic and treatment supervision, drafting the article, final approval of the version to be submitted.

Karen Redmond: editing and supervision, drafting the article, final approval of the version to be submitted.

David O'Briain: drafting the article, final approval of the version to be submitted.

## Declaration of competing interest

None.
